# Maternal mortality in the informal settlements of Nairobi city: what do we know?

**DOI:** 10.1186/1742-4755-6-6

**Published:** 2009-04-22

**Authors:** Abdhalah Kasiira Ziraba, Nyovani Madise, Samuel Mills, Catherine Kyobutungi, Alex Ezeh

**Affiliations:** 1 African Population and Health Research Center, P.O. Box 10787, 00100, Nairobi, Kenya; 2 University of Southampton, School of Social Sciences, Southampton, SO17 1BJ, UK; 3 World Bank, The World Bank, MSN G7-701, 1818 H Street NW, Washington, DC 20433, USA

## Abstract

**Background:**

Current estimates of maternal mortality ratios in Kenya are at least as high as 560 deaths per 100,000 live births. Given the pervasive poverty and lack of quality health services in slum areas, the maternal mortality situation in this setting can only be expected to be worse. With a functioning health care system, most maternal deaths are avoidable if complications are identified early. A major challenge to effective monitoring of maternal mortality in developing countries is the lack of reliable data since vital registration systems are either non-existent or under-utilized. In this paper, we estimated the burden and identified causes of maternal mortality in two slums of Nairobi City, Kenya.

**Methods:**

We used data from verbal autopsy interviews conducted on nearly all female deaths aged 15–49 years between January 2003 and December 2005 in two slum communities covered by the Nairobi Urban Health and Demographic Surveillance System (NUHDSS). In describing the distribution of maternal deaths by cause, we examined maternal and late maternal deaths according to the ICD-10 classification. Additionally we used data from a survey of health care facilities that serve residents living in the surveillance areas for 2004–2005 to examine causes of maternal death.

**Results:**

The maternal mortality ratio for the two Nairobi slums, for the period January 2003 to December 2005, was 706 maternal deaths per 100,000 live births. The major causes of maternal death were: abortion complications, hemorrhage, sepsis, eclampsia, and ruptured uterus. Only 21% of the 29 maternal deaths delivered or aborted with assistance of a health professional. The verbal autopsy tool seems to capture more abortion related deaths compared to health care facility records. Additionally, there were 22 late maternal deaths (maternal deaths between 42 days and one year of pregnancy termination) most of which were due to HIV/AIDS and anemia.

**Conclusion:**

Maternal mortality ratio is high in the slum population of Nairobi City. The Demographic Surveillance System and verbal autopsy tool may provide the much needed data on maternal mortality and its causes in developing countries. There is urgent need to address the burden of unwanted pregnancies and unsafe abortions among the urban poor. There is also need to strengthen access to HIV services alongside maternal health services since HIV/AIDS is becoming a major indirect cause of maternal deaths.

## Background

Maternal mortality, of all health indicators, exhibits the greatest disparity between the developed and developing world. Of the 536,000 deaths due to pregnancy or childbirth complications that occur each year worldwide, 96% are in Africa and Asia alone [1]. In sub-Saharan Africa where the burden is highest, the lifetime risk of dying from maternal causes is about 1 in 22 which contrasts sharply with a risk of 1 in 7,300 for women from the developed regions [1]. Over 70% of maternal deaths in developing countries are due to direct maternal causes such as hemorrhage, sepsis, hypertensive disorders, unsafe abortion, and obstructed labor. Additionally, indirect causes such as HIV/AIDS, malaria, and anemia account for about 20% of maternal deaths [2]. Given that most obstetric emergencies are often unpredictable, and that life-threatening complications occur in about 15% of all pregnancies, potentially every pregnant woman is at risk of developing a complication. Initiatives aimed at improving maternal health such as the Safe Motherhood Initiative (Nairobi, 1987) and the International Conference on Population and Development [ICPD] (Cairo, 1994) have previously been launched, but there has been little progress in improving maternal health outcomes, particularly in Sub-Saharan Africa [3]. The fifth Millennium Development Goal of reducing maternal mortality ratio by 75% between 1990 and 2015, part of the UN millennium declaration, will not be realized in many African countries if steps are not taken to reduce the prevailing high maternal mortality [1,4].

Maternal health indicators in Kenya have not improved significantly over the years. The 1998 and 2003 Kenya Demographic and Health Surveys (KDHS) recorded a national maternal mortality ratio of 590 and 414 deaths per 100,000 live births respectively while in 2005 the WHO/UNICEF/UNFPA/World Bank estimated it at 560 maternal deaths per 100,000 live births [1,5,6]. All these reports did not provide sub-national estimates which would have allowed monitoring of maternal mortality at provincial or district levels. In the 2003 KDHS, the proportion of births attended to by skilled personnel in Kenya was reported to be only 42%. There were, however, wide disparities with 79% of deliveries assisted by skilled attendants in Nairobi province as opposed to only 9% in the poorer North Eastern province [6]. Similarly, early and unplanned childbearing remain significant health and social problems in Kenya leading to many clandestine abortions [7,8]. Nearly a quarter of Kenyan women have started childbearing by age 20 and this proportion is double for women living in urban informal settlements [6,9].

Like many other health indicators, the burden of maternal mortality is heaviest among the poor [10-12]. In the context of urban informal settlements (or slums), our understanding of maternal mortality remains very limited although other indicators (such as low use of health services and increasing child mortality), suggest that the urban poor are a highly vulnerable and marginalized group [9,13]. Rapid urbanization, fueled by high levels of rural-to-urban migration, under conditions of poor economic performance has led to the growth of urban informal settlements in many African countries including Kenya. The slums are characterized by poor housing, lack of basic amenities (such as water and sanitation), and low availability and utilization of formal health services including maternity care [14,15].

There are many challenges confronting efforts to understand and address high maternal mortality burden in the developing world. Limited availability of health facilities with basic emergency obstetric care capacity, low staffing levels of birth attendants, and the lack of reliable data are a few of the many challenges. The lack of reliable data in particular, due to non-existent or largely incomplete registration of births and deaths, makes it difficult to document the magnitude of the problem and differentials across socio-economic and geographic groups [10,16]. The situation is even worse for marginalized populations such as slum residents who do not receive much attention in terms of research and/or service delivery. The presence of a population-based demographic surveillance system in the informal settlements of Nairobi provides an opportunity for accessing reasonably reliable and detailed information on maternal mortality derived from the verbal autopsy tool [17-19].

In this paper, we estimated the maternal mortality burden and identified the major causes of maternal death in two slums of Nairobi, Korogocho and Viwandani.

## Methods

### Study setting

The study was conducted in two slums in which the African Population and Health Research Center (APHRC) is implementing the longitudinal Nairobi Urban Health and Demographic Surveillance System (NUHDSS) since August 2002. The surveillance system monitors vital events such as births, deaths, and migration on over 58,000 individuals in two slum communities, Korogocho and Viwandani. The two slums are located about 5–10 km from the city centre and occupy an area less than one square kilometer in size. The main means of transport is by commuter mini-buses which drop and pick-up passengers at the periphery of the slums but do not go inside the slum settlements as there are no paved roads and there is heavy human traffic on the available paths. The informal nature of slums underscores their non-permanence with lack of public infrastructure and social services. There are very few public health facilities serving the two slum communities, and these are mainly located on the outskirts of the slums and are therefore inaccessible at night due to security concerns. The residents are from over 15 ethnic backgrounds with the majority being Kikuyu (28%), Luhya (24%), Kamba (21%) and Luo (15%). In Viwandani, the population mainly comprises labour migrants working in the neighboring industrial area, while the Korogocho population consists mainly of long-term settlers working in the informal sector.

We used data from verbal autopsies conducted on nearly all female deaths aged 15–49 years between January 1, 2003 and December 31, 2005 in the NUHDSS. We also used data from a health care facility survey conducted in 2006 to assess maternal health experiences as captured by the health management information system (HMIS) in health care facilities during 2004–2005. The health care facilities were identified from reports provided by women who participated in the household survey component of the project.

### Verbal Autopsies

As part of the NUHDSS routine procedures, verbal autopsy interviews are conducted using a questionnaire adapted from the verbal autopsy tool developed by the World Health Organization [20]. All deaths in the two slum communities are captured through a death registration form completed by a field worker. A detailed verbal autopsy interview is then conducted by a field supervisor trained to conduct verbal autopsy interviews. All verbal autopsy interviewers must have a minimum of 12 years of formal education and are familiar with the slum setting. They are trained on the verbal autopsy procedures for at least one week and retrained at the beginning of each data collection round. Interviews are conducted after making an appointment with the bereaved household normally after the funeral but within approximately 6 weeks of registering the death. Respondents are typically members of the household who cared for the deceased prior to death or have good knowledge of the symptoms or events that led to death and they must consent to be interviewed. Three physicians independently review the completed verbal autopsy forms and assign cause of death using the tenth revision of the International Classification of Diseases (ICD-10) [21]. If two or more concur, the result is then taken as the probable cause of death. Where agreement is not reached, the three physicians meet and discuss the case in order to reach a consensus. If consensus is not reached, the cause of death is coded as unknown. In this study, women who died between January 1, 2003 and December 31, 2005 were identified from the NUHDSS database. Three physicians (a medical epidemiologist and two obstetricians) independently reviewed the verbal autopsy records in order to ascertain the cause of death. The purpose of having the verbal autopsies reviewed again by two obstetricians was to ensure that any maternal deaths that could have been missed by the routine DSS coding were captured. The ICD-10 definition of maternal death, "*the death of a woman while pregnant or within 42 days of termination of pregnancy, irrespective of the duration and site of the pregnancy, from any cause related to or aggravated by the pregnancy or its management but not from accidental or incidental causes"*, was used. Further, we considered late maternal deaths defined by ICD-10 definition as *"death of a woman from direct or indirect obstetric causes more than 42 days but less than one year after termination of pregnancy" *[21]. We categorized maternal deaths into direct and indirect causes. Direct obstetric deaths are defined as *maternal deaths resulting from obstetric complications of the pregnant state (pregnancy, labor, and the puerperium), interventions, omissions, incorrect treatment or a combination of any of the above *while indirect obstetric deaths are those *resulting from previous existing disease or disease that developed during pregnancy and which was not due to direct obstetric causes, but was aggravated by physiologic effects of pregnancy *[21].

### Health Care Facility Survey

A health care facility survey was conducted in 2006 and targeted facilities that are commonly used by pregnant women living in the two slum communities for obstetric care. A total of 25 health facilities where women delivered between 2003 and 2005 were identified. Selection of health facilities was based on information provided by women who had had a pregnancy outcome between 2003 and 2005 and had been interviewed in the household survey which was part of the larger maternal health project. Some of the health facilities assessed were located in the two slums while the rest were in other parts of Nairobi.

We sought ethical approval from the Kenya Medical Research Institute (KEMRI) Ethical Review Committee, which is one of the Institutional Review Boards authorized to give ethical approval for research in Kenya. We also obtained permission from the Ministry of Health and from the Medical Officer of Health in-charge of the Nairobi City Council before visiting the health care facilities. Appointments were made with the respective health care facility personnel to explain the details of the survey after which consent was sought to carry out the interview. Structured interviews were carried out by one clinical officer who underwent three-day training for this exercise. Data on causes of deaths for 2004 and 2005 were extracted from the medical records. Descriptive statistics were used to describe the maternal mortality levels and causes of maternal death in the two slums.

## Results

During the period 2003–2005, a total of 338 women aged 15–49 years died in the two slums and verbal autopsies were completed on 289 (86%). Out of the 289 female deaths, 29 (10%) were classified as maternal deaths. Additionally, 22 (8%) deaths occurred between six weeks and one year following pregnancy termination, and were classified as late maternal deaths according to the ICD-10 definition. Table 1 shows the characteristics of maternal deaths, late maternal deaths and non-maternal deaths. Nearly three-fifths of the maternal deaths occurred in women aged 20–29 years. This sharply contrasts with 31% of non-maternal deaths in the same age category. About 18% of late maternal deaths occurred in women less than 20 years old. Over 90% of maternal deaths were of women who had at least primary level education. Of the diverse ethnic groups in the informal settlements, Luos accounted for 35% of the maternal deaths followed by the Kikuyus with 28%.

**Table 1:** Percentage distribution of maternal, late maternal and non-maternal deaths of women aged 15–49 years by socio-demographic characteristics: Nairobi Urban Health and Demographic Surveillance System, Korogocho and Viwandani, 2003–2005

	Maternal and late maternal deaths		Non-maternal female deaths
		
Characteristic	Maternal deaths	Late maternal deaths	
			
	N = 29	N = 22	N = 238
Age category			
< 20 yrs	6.9	18.2	1.7
20–29 yrs	58.6	50.0	31.1
30–39 yrs	34.5	22.7	44.5
40–49 yrs	0.0	9.1	22.7
Residence			
Korogocho	69.0	40.9	65.1
Viwandani	31.0	59.1	34.9
Ethnicity			
Kikuyu	27.6	31.8	35.7
Luo	34.5	22.7	32.8
Kamba	17.2	22.7	12.2
Luhya	13.8	22.7	12.6
Others	6.9	0.0	6.7
Education			
No education	6.9	4.6	10.5
Primary	82.8	81.8	67.2
Secondary	10.3	13.6	22.3

Table 2 presents the estimates for maternal mortality ratio using the verbal autopsy data. In computing maternal mortality ratio, cases with no data on cause of death were assumed to have a proportionate maternal mortality experience as those for which we had information on cause of death. Based on the 29 maternal deaths and adjusting for non-response, the expected number of maternal deaths was derived as shown in Table 2. Using the total number of live births of 4,806 recorded in the NUHDSS during 2003–2005, the adjusted maternal mortality ratio for this population was estimated to be 706 maternal deaths per 100,000 live births.

**Table 2:** Estimation of maternal mortality ratio, women aged 15–49 years: Nairobi Urban Health and Demographic Surveillance System, Korogocho and Viwandani, 2003–2005

	Year			
	
Characteristic/Variable	2003	2004	2005	2003–2005
Maternal deaths identified in verbal autopsies	8	10	11	29
Female deaths covered in verbal autopsies	117	100	72	289
Total female deaths	139	112	87	338
Total live births	1779	1830	1197	4806
Maternal deaths (adjusted for non-response)^1^	9.5	11.2	13.3	33.9
Unadjusted Maternal Mortality Ratio (per 100,000 live births)	450	546	919	603
Adjusted Maternal Mortality Ratio (per 100,000 live births)	534	612	1110	706

^1 ^Adjusted maternal deaths were computed as follows: (Maternal deaths/Female deaths 15–49 yrs with a VA)*All female deaths (15–49 yrs)

Table 3 shows the use of health care and pregnancy outcome. Only 14% of maternal deaths and 9% of late maternal deaths delivered or had their pregnancy terminated in a health care facility compared 65% of women had a pregnancy outcome and did not die. About 21% of maternal deaths and 32% of late maternal deaths were reported as having received skilled assistance in the process compared to 67% of women with a pregnancy outcome and did not die. Over 86% of maternal deaths and 96% late maternal deaths had sought care at least once from a professional health care worker prior to death. About 62% of maternal deaths occurred in a health care facility compared to only 31% of late maternal deaths. The pregnancy outcome of more than two-thirds of the pregnancies that ended in a maternal death was either a miscarriage or induced abortion which sharply contrasts with only 14% among the late maternal deaths and less than 2% among women who had a pregnancy outcome and did not die (Table 3). We also observed that all the abortion-rated maternal deaths in the verbal autopsy analysis had the pregnancy terminated outside of health care facility and by non-skilled personnel and less than half died in a health care facility (results not shown). This implies that the majority of abortion cases do not seek care even in the event of a complication after the abortion.

**Table 3:** Percentage distribution of maternal, late maternal deaths and women who got a pregnancy outcome but did not die by selected variables: Nairobi Urban Health and Demographic Surveillance System, Korogocho and Viwandani, 2003–2005

	Maternal and late maternal deaths		Women who had a pregnancy outcome (2003–2005) and did not die.
		
Characteristic	Maternal deaths	Late maternal deaths	
			
	N = 29	N = 22	N = 4835
Delivered by health professional			
No	79.3	68.2	33.1
Yes	20.7	31.8	66.9
Place of delivery			
Outside of health care facility	86.2	90.9	35.3
Health care facility	13.8	9.1	64.7
Outcome of pregnancy			
Abortion/still birth	69.0	13.6	1.3
Live birth	31.0	86.4	98.7
Place of death			
Outside health care facility	37.9	68.2	-
Health care facility	62.1	31.8	-
Sought care before death			
No	13.8	4.6	-
Yes	86.2	95.5	-

Table 4 shows a breakdown of the main causes of maternal death from the verbal autopsy data and from the medical records of health care facilities. Direct maternal deaths accounted for 66% of all maternal deaths while 34% of maternal deaths were due to indirect causes. The leading causes of direct maternal deaths were generally the same for the two data sources (abortion complications, antepartum and postpartum hemorrhage, postpartum sepsis, eclampsia, and ruptured uterus). The difference was the order of magnitude of the burden that each cause contributed. From the verbal autopsy data, abortion complications were the leading cause of maternal death (31%). From the medical records results, eclampsia (24%) was the leading cause of maternal mortality. For indirect causes, there were also some differences. HIV/AIDS/tuberculosis and anemia were the major indirect causes of maternal deaths as per the verbal autopsy data. Overall HIV/AIDS and tuberculosis accounted for about 59% of all late maternal deaths. Conversely, anemia and malaria were the major indirect causes as per the medical records data.

**Table 4:** Major causes of maternal mortality using data from two different sources: Nairobi Urban Health Demographic Surveillance System, (2003–05) and health care facility survey, 2006

Causes of maternal deaths from verbal autopsies in the surveillance area, 2003–2005			Causes of maternal deaths that occurred between 2004 and 2005. Health Care Facility Survey 2006,		
*Direct maternal causes*	*Number*	*Percentage (%)*	*Direct maternal causes*	*Number*	*Percentage (%)*

Abortion related	9	31.0	Abortion related	23	7.3
Ante/post partum hemorrhage	4	13.8	Ante/postpartum hemorrhage	33	10.4
Postpartum sepsis	3	10.3	Postpartum sepsis	43	13.6
Eclampsia	2	6.9	Eclampsia	75	23.7
Ruptured uterus	1	3.5	Ruptured uterus	13	4.1
			Retained placenta	10	3.2
			Prolonged labor	5	1.6
			Ectopic pregnancy	3	0.9
			Other direct	4	1.3

*Indirect maternal causes*			*Indirect maternal causes*		
HIV/AIDS/TB	4	13.8	TB/HIV/AIDS	9	2.8
Anemia	2	6.9	Anemia	39	12.3
Other indirect maternal causes	4	13.8	Other indirect maternal causes	32	10.1
			Malaria	27	8.5
Total	29	100.0	Total	316	100.0

## Discussion

We estimated maternal mortality burden in two Nairobi slums and showed that the maternal mortality ratio (706/100,000) is higher than the national estimates (560/100,000). Using the two data sources we also identified the leading causes of maternal death as: abortion complications, eclampsia, postpartum sepsis, hemorrhage, and ruptured uterus. The order of magnitude each cause contributes differs for the two data sources. Abortion complications were the most important cause of maternal mortality in the population-based approach. It is not clear what could explain this observation. We speculate that it might be an issue of diagnosis ascertainment bias in the verbal autopsy data or incompleteness of health care facility records. For instance abortion is restricted in Kenya and hence likely to be under-reported in health facilities. It is also important to note that the larger health facilities have a wider catchment area therefore it is possible that the distribution of causes of maternal deaths is different from that of the slums. These differences notwithstanding, our results generally concur with what is generally known to be the major causes of maternal mortality in sub-Saharan Africa [22-24]. In spite of 47% of the health care facilities providing incomplete records, results from the health care facilities are consistent with other hospital based studies that have shown eclampsia and hemorrhage as the leading causes of maternal mortality.

Current evidence suggests that maternal mortality in developing countries could be reduced if all pregnant women could have access to health professionals as well as quality emergency obstetric care services [25-27]. In this study most deliveries and abortions occurred outside of a health care facility and without professional assistance. An earlier study from the same population over the same period initially estimated that 70% of women interviewed delivered with professional assistance but on closer scrutiny of who actually attended the deliveries, it was shown that only 48% delivered with the assistance of skilled birth attendants [28]. In this study, only 21% of all maternal deaths delivered with the help of a skilled attendant compared to 67% of women with a pregnancy outcome and did not die. This is an indication that indeed most maternal deaths happened following mismanaged labor or abortion by non-skilled attendants. All abortions related deaths followed an abortion carried by a non-professional and less than 50% sought care before death. Given this scenario, it is likely that hospital-based studies using medical records miss out on abortions especially clandestine ones in countries such as Kenya where abortion is illegal [29]. Alternatively, given the legal restrictions on induced abortion, health facilities could be deliberately not recording abortion as a cause of death.

The differences in the order of magnitude of the indirect causes of maternal mortality could be due to a number of factors. From the verbal autopsy data, HIV/AIDS/Tuberculosis and anemia were the major causes compared to anemia and malaria presented in the health care facility records. The low number of deaths attributed to HIV/AIDS in the health facilities was surprising given the high AIDS-related mortality in this population [30]. HIV/AIDS is increasingly contributing to the burden of pregnancy related deaths [31,32] and it is not clear whether the differences were due to measurement errors whereby the verbal autopsy tool over diagnosed HIV/AIDS and the health care facility over diagnosed malaria. Verbal autopsy coders could have over diagnosed HIV/AIDS especially for cases where vague signs and symptoms were reported given their knowledge that HIV/AIDS is prevalent and a major cause of adult deaths in this population. Is it also possible that HIV/AIDS in health care facilities may largely be reported as an underlying cause and what gets reported in the health care facility records are the immediate causes. On the other hand most cases labeled as having died from malaria in health care facilities are not confirmed by laboratory investigations and thus diagnoses are mainly based on history and clinical examination. Given that Nairobi is a low malaria transmission area, it is likely that those labeled as having malaria might have died from other febrile causes.

Whereas most deliveries and abortions that resulted into a maternal death took place outside of a health care facility, most of them died at a health care facility. This might be a pointer to delays in recognizing a complication, making a decision to go to a health care facility, and reaching it or receiving care while at the facility. The lack of emergency ambulance services in the slums where infrastructure is non-existent and insecurity deters movement at night further complicates referral. It may as well be that the quality of emergency obstetric services is poor, an observation also reflected in the high proportion of deaths due to puerperal sepsis. An earlier health care facility assessment study showed that more than half of obstetric emergencies arrive at referral facilities either on foot or by public means and that emergency obstetric care facilities are lacking in staffing, skills, and equipment [33].

The verbal autopsy approach which takes advantage of the existing surveillance system circumvents the challenges of selection bias which hospital-based studies face since many maternal deaths in this community occur at home. In such settings, the verbal autopsy approach can be used to identify both health care facility and community-based maternal deaths [34,35] to improve our understanding of the problem. Verbal autopsy interviews, however, may suffer from the risk of recall bias and possible misclassification of the cause of death due to wrong recording and misinterpretation of signs and symptoms.

### Limitations

The small numbers of maternal deaths from the verbal autopsy data presents a challenge in trusting the estimates and presents a common limitation with maternal mortality estimates [6,16]. Up to 14% of all female deaths did not have a verbal autopsy and about 47% of the health care facility medical records were incomplete. This could have potentially introduced bias in the estimates. The national estimate in KDHD 2003 was not disaggregated by province or rural-urban residence making comparison with the estimate found in the Nairobi slums difficult. Due the incomplete health care facility records and lack of proper catchment population and hence denominator, the health care facility survey data could not be used to estimate maternal mortality ratio. However, these data were used to establish the leading causes of maternal deaths.

## Conclusion

Maternal mortality ratio is high in the slum population. The leading causes of maternal death are: abortion complications, eclampsia, hemorrhage, sepsis and ruptured uterus. There is urgent need to address the burden of unwanted pregnancies and unsafe abortions among the urban poor and to promote programs that facilitate access to contraceptives to mitigate the occurrence of unwanted pregnancies and unsafe abortions. Most maternal deaths occur to women who deliver outside of a health care facility and with the help of unskilled personnel. Efforts should be made to ensure that all deliveries occur with help of skilled personnel. HIV/AIDS is becoming a major indirect cause of maternal death and often occur after the traditional 42-day cut-off period used to define maternal death. There is therefore need to strengthen access to HIV services alongside maternal health services. In an environment with scarcity of reliable data, different research approaches and sources of data can help fill the existing data gaps. We have demonstrated this by showing that the verbal autopsy tool seem to capture abortions better than facility based records and can be a good additional source of data for establishing the burden of maternal mortality attributable to abortion complications.

## Competing interests

The authors declare that they have no competing interests.

## Authors' contributions

AKZ took lead in preparing the manuscript. He participated in data collection, data cleaning, and conducted most of the analysis. NM conceptualized the idea of this paper; she supervised data collection and analysis and wrote sections of this paper. SM contributed to data analysis, manuscript preparation, and interpretation of findings. CK contributed to manuscript preparation and interpretation of findings. AE contributed to the conceptualization of the study, manuscript preparation, and interpretation of findings.
